# Lung India in PubMed

**DOI:** 10.4103/0970-2113.63602

**Published:** 2010

**Authors:** S. K. Jindal

**Affiliations:** *Chief Editor, Lung India, E-mail: skjindal@indiachest.org*

The New Year 2010 brings cheers to the readers of Lung India and all other members of the Indian Chest Society. The journal has now been selected for indexing by PubMed.[1] This has provided a great opportunity for those who contribute to Lung India to have their research and other scientific content exposed to people worldwide. Scientists, clinicians and others working in different areas in different countries at different places can now browse through the contents of published information in our journal. It is more important for us to expose ourselves and our work to the wide scientific world, face criticism and continually attempt to improve and improvise.

Lung India not only interests the Indian fraternity, but also it serves as a source for those outside India to have a few glimpses of pulmonary practice in India. It may not have an impact factor (IF) as yet, but it does impact the overall healthcare systems and sciences in the country.[2] In spite of the great value of the IF for the assessment of a journal, the fact cannot be denied that its value is limited in the context of the importance of a journal in the overall scientific scene.[2] It also cannot be denied that several international journals have tended to manipulate an artificial increase in IF.[3] Therefore, people have tended to adopt other criteria and indices such as the total circulation and subscription, submission and rejection rates, etc.

I am also glad to say that in addition to PubMed, the journal is also indexed/listed with Caspur, DOAJ, EBSCO Publishing's Electronic Databases, Excerpta Medica/ EMBASE, Expanded Academic ASAP, Genamics JournalSeek, Global Health, Google Scholar, Health and Wellness Research Center, Health Reference Center Academic, Hinari, Index, Copernicus, Index Medicus for South-East Asia Region, Indian Science Abstracts, IndMed, MANTIS, MedInd, OpenJGate, SCOLOAR, SIIC databases and Ulrich's International Periodical Directory.

All the achievements also put a great degree of onus and responsibility on our shoulders. We all need to participate in the continued growth of the journal. It is important that we achieve a higher standard of medical writing. It is a consistent observation that many of our authors do not stick to the rigid criteria of writing manuscripts and take adequate care of the contents, the grammar and the reference citation. We need to contribute more of our research findings and observations than mere care-reports, reviews and C.M.E.s. Tremendous inputs are required to improve our quality. We also need to make our reviewing system as more efficient, date-bound and effective. This shall help in our long-term goals to achieve greater heights.

Lastly, I wish to convey my thanks to the readers of Lung India who have borne with me for the last several years. I am sure that the new Editorial Board which soon takes over shall expand and improve the scope of the journal. I of course shall continue to contribute my might.
